# Hookworm Prevalence in England

**Published:** 1920-11-20

**Authors:** 


### A CARRIER QUESTION.

### Hookworm Prevalence in England.

To the student of tropical and sub-tropical medicine there is no great novelty in the more recent history of the condition known colloquially as hookworm disease, and more accurately as ankylostomiasis. The menace that this infection holds for white races in the less civilised parts of Australia has been frequently referred to in these pages among the medical notes from that continent, and the prevalence in sundry home mining districts is a well-known local trouble. But recent inquiry among practitioners in various parts of this country elicits the fact that in a number of instances unsuspected cases of hookworm infection are being brought to light, and since it is, in the majority of instances, an utterly unsuspected infection there seems to be every reason to draw attention to its possible presence in the *rôle* of a complication capable of intensifying a variety of other disorders.

Most of the war and post-war diseases from which the country was in danger through the "carrier" agency have received such insistent public notice that we have undoubtedly grown to keep a reasonably sharp look-out for them. But from personal experience the writer can vouch for the fact that a number of hookworm cases seem to have escaped recognition.

### The Overseas Origin.

Without dwelling long upon the etiological side of the disease, it may be recalled that ankylostomiasis is very prevalent in Egypt, and a considerable percentage of white troops were known to be infected. Amongst Indian troops, particularly in Palestine, East Africa, and Mesopotamia, outbreaks at times produced serious disability and indirectly infected a number of whites associated with them. But the peculiar circumstances of the infection thus established come with the fact that the parasite may often exist without any noticeable immediate injury to the carrier's health, or at most may produce a certain amount of debility and loss of weight until some other malady tends to lower the vital resistance and the combined effect produces definite illness. If suspected, the disease is easily recognised by finding the ova in the stools, but in the average home practice the chances of altogether missing the hookworm infection are obvious.

As an example, there is evidence to show that adult carriers are curiously liable to infection with measles, though they may have had this disease in earlier years. But the types of common and well-recognised English sickness which ankylostomiasis may simulate will be best appreciated from an outline of the actual mode of the parasite's existence. Male and female forms of these small cylindrical worms inhabit, especially the jejunum, though they sometimes stray as far as the duodenum above or the ileum below. Attaching themselves to the mucous membrane, they live by a process of drawing blood from the vessels thereof, and the symptoms they produce are partly due to very gradual loss of blood, partly to the slow destruction of the mucosa over the feeding areas. Also the bodies of the parasites undoubtedly contain hæmolytic substances, and it is suggested that some of these may find their way into the carrier's system. Obviously in alimentary areas irritated by such activities secondary bacterial infections may readily occur.

### The Mode of Transference.

As with all these infections, the egg of the parasite is passed by the carrier per rectum, and hatches out under moist warm conditions, but in the mode of re-entry to a human host the great novelty lies. In twenty-four hours, if circumstances are favourable, the young embryo can be seen coiled up in its egg. In two to seven days it is hatched out, and as a minute larva moults several times, grows larger, and makes for moist earth or wood or some small pool. At this stage it is active and can swim, and even climb up any wet surface, a fact which explains the situation of certain skin eruptions which are seen to occur in the disease. At this stage reinfection is effected. Entry to a new host is made in one of two ways; through the mouth or through the skin. Surprisingly perhaps, the latter is the more frequent, although contamination of drinking water, food, vegetables, etc., is a possibility, and, naturally, if taken by the mouth, the route of the jejunum is simple.

### A Remarkable Journey.

The skin route, however, is by way of the hair follicles, through which the larvæ pass, and from the subcutaneous tissues they attain veins and lymphatics, and travel gradually to the intestine *viâ* the heart, pulmonary vessels, air-cells of the lungs, bronchi, trachea, larynx, and œsophagus to the stomach. This extraordinary journey is believed to take about eight days. Once in the jejunum the reproductive cycle begins again, but the point of importance in this country, among reasonably clean-living people, is that, although we do not know exactly how long one set of parasites may live in any particular carrier, it is undoubtedly a considerable time. Also that reinfection can on occasion occur will be appreciated from common experience of other helminthic diseases.

But to return to those symptoms commonly seen; as might be expected, they depend directly upon the number of parasites present. Presumably in any carrier case there will be relatively few. Other factors, too, must be recognised, namely, general physique, personal idiosyncracy to the toxins, and the presence of associated diseases. In the tropics opinions vary as to which plays the greatest part, but undoubtedly in the returned traveller we have merely to look out for a more or less severe

**A Carrier Question**—(*continued*).

secondary anæmia. In this cryptic type there is commonly slight digestive trouble, with tenderness, pain, or discomfort in the epigastrium. Often there is a slight reduction in the hæmoglobin in the blood and a small, but noticeable, loss in the power of mental concentration. A blood examination may show the customary eosinophilia, and it is often said that such patients do not realise they have been ill—in the sense of a specific disease—until they have been cured.

Here it is needless to recount in detail the train of more severe but corresponding symptoms which follow a heavier infection, since such is not the type of case likely to be "missed" by either the official or private medical man through whose hands the patient may pass. In brief, they are physical and mental fatigue, various nervous symptoms and joint pains, palpitation, dyspnœa, and a persistent œdema, so that the patient develops a remarkable resemblance to a man suffering from chronic nephritis. In the tropics anæmia *plus* dropsy always leads one to think of ankylostomiasis, but to us, in England, so advanced a state is naturally rare, and we have simply to combat the possibility that a set of simple troubles as outlined above *may* have a more definite and localised cause than common experience would suggest.

On the other hand, the treatment of such mild cases by vermifuges is satisfactory, if carefully controlled, and the details call for no particular comment. The main importance attaches to the existence of the type of carrier case in relatively unsuspected quarters. The risk of infection of others, particularly in rural districts, is obvious, and in conclusion it is necessary only to note a simple method by which any medical man with a low-powered microscope can detect the excreted ova.

Dry microscopical preparations are useless. A coverglass need not be used. The eggs are covered with a thin gelatinous coating, so that, if a small portion of washed and sedimented fæces is placed in the centre of a slide, and the whole very gently immersed in water, the ova remain after everything else has floated off. If this process is repeated several times on the same area of the slide, quite a number of the eggs are often collected close together. Of their identification we need not remark as the characters are well appreciated and illustrated in the standard works. It will suffice that attention has been drawn to the fact that these hookworm cases do occur, and not so very infrequently, in British practice to-day, and that a sketch has been given, brief though it is, of the circumstances and means of their recognition.

